# Macromolecular crystallography using microcrystal electron diffraction

**DOI:** 10.1107/S2059798320016368

**Published:** 2021-02-17

**Authors:** Max T. B. Clabbers, Hongyi Xu

**Affiliations:** a Department of Materials and Environmental Chemistry, Stockholm University, 106 91 Stockholm, Sweden

**Keywords:** electron crystallography, 3D electron diffraction, microcrystal electron diffraction, macromolecular crystallography, methods development

## Abstract

Microcrystal electron diffraction (MicroED) has recently emerged as a promising method for macromolecular structure determination in structural biology. Since the first protein structure was determined in 2013, the method has been evolving rapidly. Several protein structures have been determined and various studies indicate that MicroED is capable of (i) revealing atomic structures with charges, (ii) solving new protein structures by molecular replacement, (iii) visualizing ligand-binding interactions and (iv) determining membrane-protein structures from microcrystals embedded in lipidic mesophases. However, further development and optimization is required to make MicroED experiments more accurate and more accessible to the structural biology community. Here, we provide an overview of the current status of the field, and highlight the ongoing development, to provide an indication of where the field may be going in the coming years. We anticipate that MicroED will become a robust method for macromolecular structure determination, complementing existing methods in structural biology.

## Abstract 2

The current status and ongoing development of 3D electron diffraction and microcrystal electron diffraction in macromolecular crystallography are reviewed.

## 1. Introduction   

For the past several decades, X-ray crystallography has been the most prominent method for protein structure determination in structural biology. However, the rate-limiting step in macromolecular crystallography inhibiting structure determination is often crystallization (Terwilliger *et al.*, 2009 ▸; Luft *et al.*, 2011 ▸). Growing crystals of sufficient size and order can be challenging, especially in the case of membrane proteins (Carpenter *et al.*, 2008 ▸; Caffrey, 2003 ▸). Large and well ordered crystals are required to provide sufficient structural information before radiation damage deteriorates the crystallinity and data quality. Therefore, macromolecular crystals of at least several micrometres in size are typically required for structure determination via conventional single-crystal X-ray crystallo­graphy (Nave & Hill, 2005 ▸; Holton & Frankel, 2010 ▸; Sanishvili *et al.*, 2011 ▸).

Whereas crystallization trials that failed to grow crystals for X-ray diffraction are often discarded, experimental evidence indicates that these often contain small macromolecular crystals (Calero *et al.*, 2014 ▸; Stevenson *et al.*, 2014 ▸, 2016 ▸). Such (sub-)micrometre-sized protein crystals can still be useful for structure determination, and the small crystal volume offers certain advantages over larger counterparts. Notably, microcrystals may be better ordered and have fewer defects compared with larger crystals (Cusack *et al.*, 1998 ▸; de la Cruz *et al.*, 2017 ▸; Wolff *et al.*, 2020 ▸). Other advantages include rapid flash-cooling during vitrification, preserving the protein crystal in its native hydrated state and limiting evaporation, as well as fast and uniform soaking via efficient diffusion of ligands or heavy metals into the microcrystals (Clabbers *et al.*, 2020 ▸; Martynowycz & Gonen, 2020 ▸). However, one major dis­advantage of using microcrystals is radiation damage, which limits the diffracting intensity and attainable resolution (Hattne *et al.*, 2018 ▸).

Serial femtosecond crystallography (SFX) can make use of such small protein microcrystals by outrunning the radiation damage using a diffraction-before-destruction scheme. SFX data are collected using an X-ray free-electron laser (XFEL), which generates brief and highly intense X-ray pulses on a femtosecond timescale, taking single still diffraction snapshots of partial reflections from individual hydrated microcrystals before the sample is destroyed (Schlichting, 2015 ▸; Spence, 2017 ▸). SFX enables fast time-resolved studies at room temperature, and the measured intensities are accurate enough for *de novo* phasing (Barends *et al.*, 2014 ▸). The downsides of SFX are the associated costs and limited access to large-scale XFEL facilities, and the relatively large amount of sample required. Typically, data from several thousand still diffraction patterns need to be merged to obtain a complete diffraction data set that is suitable for structure determination.

Electrons interact strongly with matter and are less damaging than X-rays per elastic scattering event by several orders of magnitude (Henderson, 1995 ▸). The mild radiation damage relative to the useful kinematic signal that electrons offer makes them an alternative for macromolecular structure determination. In electron crystallography, high-energy electrons are accelerated in vacuum by an electron gun or filament to form a (coherent) electron beam in a transmission electron microscope (TEM). When operated in diffraction mode, the diffracted intensities of electrons scattered by the crystal are measured but lack the phase information. In imaging mode, real-space images are recorded and the spatial phase information is retained. In 2D electron crystallography, a 3D reconstruction of the protein structure can be obtained from 2D projection images recorded at different tilt angles by combining diffraction intensities extracted from electron diffraction patterns and crystallographic structure-factor phases extracted from electron micrographs (De Rosier & Klug, 1968 ▸). Using this reconstruction method, several protein structures were solved from thin 2D crystals (Unwin & Henderson, 1975 ▸; Henderson *et al.*, 1990 ▸; Grigorieff *et al.*, 1996 ▸; Mitsuoka *et al.*, 1999 ▸; Gonen *et al.*, 2005 ▸). Initial low-resolution phase information extracted from the micrographs can be extended by using high-resolution diffraction patterns for structure determination (Wisedchaisri & Gonen, 2011 ▸). Traditionally, radiation damage has severely limited the success of protein structure determination from 2D crystals (Dorset & Parsons, 1975 ▸; Unwin & Henderson, 1975 ▸; Glaeser & Downing, 1993 ▸). Furthermore, it is challenging to grow a perfect single-crystalline 2D layer of proteins devoid of any defects or distortions. These effects can be mitigated by the use of improved detectors and processing algorithms adapted from cryo-EM single-particle imaging (Righetto *et al.*, 2019 ▸).

Single-particle cryo-EM has recently made a great impact on structural biology (Kühlbrandt, 2014 ▸; Cheng, 2018 ▸). Here, projections of individual protein molecules from electron micrographs are averaged in 2D and reconstructed into a 3D real-space volume (De Rosier & Klug, 1968 ▸; Cheng *et al.*, 2015 ▸). The crystallization step is thus avoided in single-particle analysis (SPA). Using SPA, many protein structures could be determined from high-resolution images (Bartesaghi *et al.*, 2015 ▸; Fischer *et al.*, 2015 ▸; Merk *et al.*, 2016 ▸), recently even pushing the structure determination up to atomic resolution (Yip *et al.*, 2020 ▸; Nakane *et al.*, 2020 ▸). However, such high-resolution reconstructions are often from larger and rigid proteins or protein complexes. It becomes increasingly challenging to obtain sufficient contrast from cryo-EM imaging for protein complexes smaller than 50 kDa (Henderson, 1995 ▸; Glaeser, 1999 ▸; Khoshouei *et al.*, 2017 ▸; Fan *et al.*, 2019 ▸; Herzik *et al.*, 2019 ▸).

## 2. Microcrystal electron diffraction   

### 2.1. Electron diffraction data collection   

**Figure 1:**
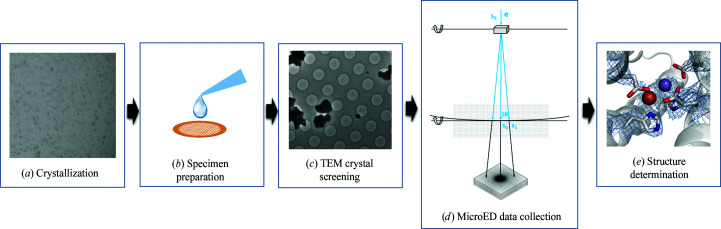
Schematic overview of a typical workflow involved in MicroED. (*a*) Microcrystals are grown using sitting-drop vapour diffusion. When a crystallization drop is identified containing potential microcrystals suitable for MicroED, the drop is mixed with buffer solution and (*b*) deposited on a standard EM grid. Excess liquid is blotted away using filter paper, either from the back-side or both sides of the grid, and the grid is vitrified and cryo-transferred to the TEM. (*c*) In imaging mode, the grid can be screened for thin hydrated protein microcrystals that are suitable for data collection. (*d*) Switching to diffraction mode, MicroED data can be collected by continuously rotating the microcrystal about the rotation axis, effectively rotating the crystal lattice in reciprocal space, analogous to the rotation method in macromolecular X-ray crystallography. The diffraction patterns can then be indexed, the intensities are extracted and the structure can be determined by molecular replacement (*e*) followed by model building and structure refinement.

In electron diffraction, structural information from 3D (sub-)micrometre-sized crystals can be obtained from proteins that are well below 50 kDa (Nannenga & Gonen, 2019 ▸), even down to short peptide fragments (Rodriguez *et al.*, 2015 ▸; Sawaya *et al.*, 2016 ▸). The signal is significantly boosted by having a crystalline ordered array of protein molecules. Electron diffraction data of protein crystals can effectively be collected by (continuously) rotating the crystal about a single rotation axis (Nederlof, van Genderen *et al.*, 2013 ▸; Nannenga, Shi, Leslie *et al.*, 2014 ▸), analogous to the rotation method in X-ray crystallography (Arndt & Wonacott, 1977 ▸; Dauter, 1999 ▸) and to related existing 3D electron diffraction (3DED) data-collection strategies in TEM (Kolb *et al.*, 2008 ▸; Wan *et al.*, 2013 ▸; Gemmi *et al.*, 2019 ▸). In 2013, the first electron diffraction rotation series was acquired from 3D protein nanocrystals (Nederlof, van Genderen *et al.*, 2013 ▸), and soon after the first protein structure was determined by stepwise and subsequently continuous rotation using microcrystal electron diffraction (MicroED; Shi *et al.*, 2013 ▸; Nannenga, Shi, Leslie *et al.*, 2014 ▸; Fig. 1 ▸). For some microscopes, owing to poor mechanical alignment of the goniometer, the crystal may drift in the *x*, *y* and *z* directions during continuous-rotation MicroED data collection. Movement in the *x* and *y* directions will cause the crystal to move out of the electron beam, while displacement in the height *z* will cause inaccuracy in unit-cell determination. We recommend selecting crystals near the centre of the grid and aligning each crystal to the physical eucentric height before data collection (Yonekura *et al.*, 2015 ▸; Shi *et al.*, 2016 ▸; Zhou, Luo, Luo *et al.*, 2019 ▸). New software and hardware development is under way to overcome this issue. For example, the software *Instamatic* allows crystal tracking during continuous-rotation data collection by defocusing the diffraction beam to form a ‘shadow’ image of the crystal once every few frames (Smeets *et al.*, 2018 ▸).

To limit radiation damage, diffraction data typically need to be collected rapidly and under low-dose conditions. For hydrated protein crystals exposed to the electron beam, half of the mean diffracted intensities of reflections is lost after a dose of about 2.2 e^−^ Å^−2^ (Hattne *et al.*, 2018 ▸). Furthermore, radiation damage induces site-specific loss of structural features such as metal cofactors, breakage of disulfides and decarboxylation of acidic side chains, which will deteriorate map and model quality. As electron scattering is affected by the charged states of atoms, especially at low resolution, metal ions and charged side chains may be particularly susceptible to irradiation (Yonekura & Maki-Yonekura, 2016 ▸; Yonekura *et al.*, 2018 ▸). Until recently, radiation damage limited data collection to only a few still diffraction patterns (Dorset & Parsons, 1975 ▸; Unwin & Henderson, 1975 ▸; Glaeser & Downing, 1993 ▸; Georgieva *et al.*, 2011 ▸). One of the decisive advances that made it feasible to record (continuous) rotation data was the introduction of complementary metal oxide semiconductor (CMOS) sensors, hybrid pixel detectors (HPDs) and direct electron detectors (DEDs), which enabled rapid data collection and highly accurate measurements, even at low exposures of typically 0.1–0.01 e^−^ Å^−2^ s^−1^ (Georgieva *et al.*, 2011 ▸; Nederlof, van Genderen *et al.*, 2013 ▸; Nannenga, Shi, Leslie *et al.*, 2014 ▸; Hattne *et al.*, 2016 ▸, 2019 ▸; Shi *et al.*, 2016 ▸; Clabbers *et al.*, 2017 ▸).

### 2.2. Data processing   

The similarities to macromolecular X-ray crystallography enable most programs and processing routines to be adapted to process electron diffraction data with only minor modifications. Diffraction data are preferably collected with an oscillation width that is smaller than the rocking curve of a typical reflection, generally less than 1.0° per frame. Fine-slicing reduces background noise by recording partial reflections and thus sampling the reflection profile over several adjacent frames (Mueller *et al.*, 2012 ▸). This also enables 3D profile fitting to extract weak or even negative intensities below the noise level (French & Wilson, 1978 ▸; Oatley & French, 1982 ▸; Pflugrath, 1999 ▸; Leslie, 1999 ▸). Electron diffraction data can be processed using profile fitting in, for example, *XDS* (Kabsch, 2010 ▸), *MOSFLM* (Leslie, 2006 ▸) and *DIALS* (Winter *et al.*, 2018 ▸; Clabbers *et al.*, 2018 ▸). The much shorter wavelength of high-energy electrons (0.0251–0.0197 Å for 200–300 keV electrons) compared with X-ray diffraction (1.0332 Å for 12 keV X-ray photons) does however affect the diffraction geometry. Notably, the Ewald reconstruction is virtually flat for electron diffraction (Fig. 1 ▸). Reflections from higher-order Laue zones are typically not observed, and Friedel pairs can sometimes both be measured on the same frame.

### 2.3. Structure determination   

**Figure 2:**
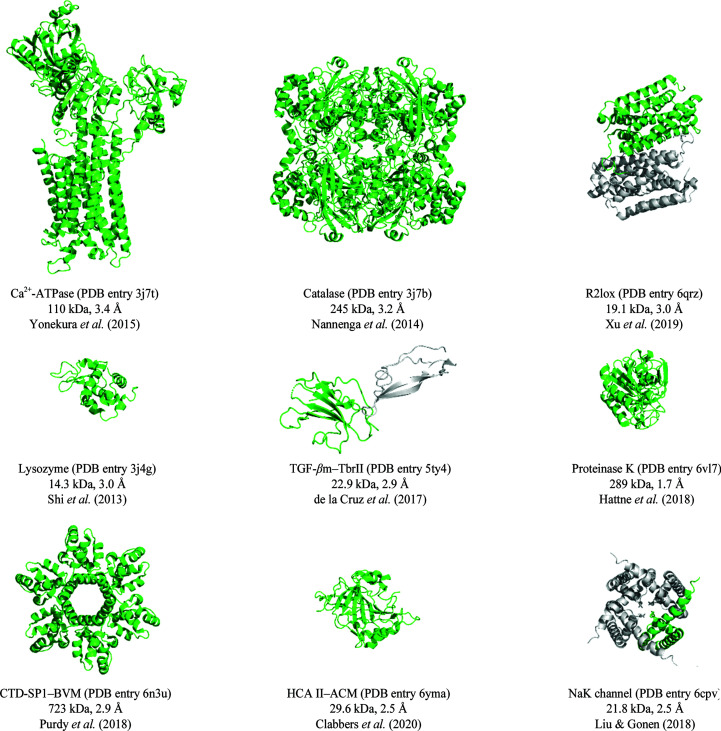
Highlighting several protein structures determined by MicroED. Figures were prepared using the *PyMOL* molecular-graphics system version 2.2.3 (Schrödinger).

Since the first protein structure of tetragonal hen egg-white lysozyme (HEWL) was determined in 2013 (Shi *et al.*, 2013 ▸; Nannenga, Shi, Leslie *et al.*, 2014 ▸), several other protein structures have successfully been determined using MicroED (Fig. 2 ▸; Nannenga, Shi, Hattne *et al.*, 2014 ▸; Yonekura *et al.*, 2015 ▸; de la Cruz *et al.*, 2017 ▸; Clabbers *et al.*, 2017 ▸, 2020 ▸; Hattne *et al.*, 2018 ▸; Purdy *et al.*, 2018 ▸; Xu *et al.*, 2018 ▸, 2019 ▸; Liu & Gonen, 2018 ▸). In all of these cases phases were obtained via molecular replacement (Rossmann, 1990 ▸; Vagin & Teplyakov, 2010 ▸; McCoy *et al.*, 2007 ▸), and the vast majority of these proteins are of known structures and unit-cell dimensions as previously determined using X-ray diffraction. A rare ortho­rhombic crystal polymorph of dimeric HEWL has recently been reported (Clabbers *et al.*, 2017 ▸; Xu *et al.*, 2018 ▸), and a previously unobserved monoclinic crystal lattice of HEWL was subsequently discovered (Lanza *et al.*, 2019 ▸); however, both were phased using a previously determined structure of the identical protein. Only recently, a novel protein structure was solved by MicroED of an unknown metalloenzyme, R2lox, with unknown unit-cell dimensions. The structure could be solved by molecular replacement using a search model of only 35% sequence similarity to its closest known homologue (Xu *et al.*, 2019 ▸). For structure determination, standard crystallo­graphic routines can be applied for MicroED data in the *CCP*4 (Winn *et al.*, 2011 ▸) and *Phenix* (Liebschner *et al.*, 2019 ▸) software suites, including structure solution (Vagin & Teplyakov, 2010 ▸; McCoy *et al.*, 2007 ▸), model building (Emsley *et al.*, 2010 ▸; Croll, 2018 ▸) and refinement (Murshudov *et al.*, 2011 ▸; Afonine *et al.*, 2012 ▸). We note that electron scattering factors are available in these software suites and they should be used for MicroED data.

### 2.4. Membrane proteins   

Structural models of membrane proteins have successfully been determined in the past using MicroED from 3D microcrystals of the Ca^2+^-dependent ATPase (Yonekura *et al.*, 2015 ▸) and an NaK ion channel (Liu & Gonen, 2018 ▸) (Fig. 2 ▸). Membrane proteins are often notoriously difficult to crystallize, preferring a native lipidic environment. In 2D electron crystallography, membrane proteins are embedded in a lipid bilayer mimicking a more natural environment (Unwin & Henderson, 1975 ▸; Glaeser & Downing, 1993 ▸). Alternatively, lipidic cubic phase (LCP) has been demonstrated to be an effective way to grow 3D crystals of membrane proteins (Landau & Rosenbusch, 1996 ▸; Caffrey, 2003 ▸; Cherezov, 2011 ▸). Unfortunately, the high viscosity of LCP makes the TEM sample preparation highly challenging and has complicated structure determination (see also Section 3.3). By inducing a transition from LCP to less viscous mesophases or sponge phases, it has recently been illustrated that LCP-grown microcrystals of G protein-coupled receptor (GPCR) could be analysed by MicroED (Zhu *et al.*, 2020 ▸). An alternative way to deal with the high viscosity is by using cryo-focused ion-beam (cryo-FIB) milling to prepare thin crystalline lamella (see also Section 3.4), which has been demonstrated as a potential routine for lipid-embedded bacteriorhodopsin microcrystals (Polovinkin *et al.*, 2020 ▸). However, these reports did not resolve a structural model of any lipid-embedded membrane protein. Recently, using optimized sample preparation and cryo-FIB milling, the structure of a lipid-embedded voltage-dependent anion channel was determined using MicroED (Martynowycz, Khan *et al.*, 2020 ▸). Soon after, the structure of a GPCR was successfully determined by converting LCP into a less viscous sponge-like phase combined with cryo-FIB milling (Martynowycz, Shiriaeva *et al.*, 2020 ▸). These results demonstrate that many potentially difficult-to-crystallize membrane proteins embedded in lipid mesophases can become targets for structure determination by MicroED.

### 2.5. Drug discovery and fragment screening   

Another potential application of electron diffraction is in drug discovery via structure-guided drug design and fragment-based screening, which is an important discipline in structural biology (Blundell *et al.*, 2002 ▸; Hajduk & Greer, 2007 ▸). MicroED may have some advantages for fragment screening as data can rapidly be collected in-house on a conventional TEM (Zhou, Luo, Luo *et al.*, 2019 ▸; Clabbers *et al.*, 2020 ▸). Furthermore, ligands can be efficiently soaked into the microcrystals owing to rapid diffusion into small volumes (Martynowycz & Gonen, 2020 ▸; Clabbers *et al.*, 2020 ▸). Early work on resolving drug-binding interactions using MicroED involved the binding of the inhibitor bevirimat (BVM) to the C-terminal domain of the HIV Gag fragment (Purdy *et al.*, 2018 ▸; Fig. 2 ▸). However, although the structural model provided insight into the underlying interactions in inhibitor binding, a unique binding pose could not be assigned based on the MicroED data alone (Purdy *et al.*, 2018 ▸). This was likely to have been complicated by the moderate resolution of 2.9 Å and the location of the binding site positioned on the symmetry axis at the centre of the multimeric complex (Fig. 2 ▸). Recently, drug-binding interactions were successfully visualized using MicroED from microcrystals of human carbonic anhydrase isoform II (HCA II) complexed with the clinical drug acetazolamide (AZM; Clabbers *et al.*, 2020 ▸; Fig. 2 ▸). The ligand could successfully be resolved from the difference potential map at 2.5 Å resolution. These studies demonstrate the potential for screening ligand-binding interactions using MicroED. However, data accuracy and precision, as well as resolution, will likely need to be improved before it can become a viable method for structure-guided drug design.

## 3. Crystallization and sample preparation   

### 3.1. Crystal size   

One of the major advantages of MicroED is that it significantly eases the requirements on crystal size which have been challenging in macromolecular crystallography. The ideal crystal size and morphology for electron diffraction experiments is dependent on several factors. Multiple elastic scattering events (dynamical scattering) occur more frequently with increasing crystal thickness and will affect structure determination (Cowley, 1995 ▸; Dorset, 1995 ▸). This dictates the use of thin hydrated protein crystals, preferably in the range of about 100–200 nm, to reduce the effects of dynamical scattering, depending on the electron energy (Dorset & Parsons, 1975 ▸; Glaeser & Downing, 1993 ▸; Dorset, 1995 ▸; Subramanian *et al.*, 2015 ▸; Clabbers & Abrahams, 2018 ▸; Latychevskaia & Abrahams, 2019 ▸). However, several protein structures could successfully be determined from crystals that were substantially thicker. In fact, there is a trade-off in reducing dynamic scattering and optimizing the signal-to-noise ratio, as an increasing number of unit cells will boost the diffracting signal significantly.

### 3.2. Crystallization   

Growing small microcrystals for MicroED is feasible using standard routines, optimizing the crystallization conditions using sitting-drop or hanging-drop vapour diffusion (Georgieva *et al.*, 2007 ▸; Nederlof *et al.*, 2011 ▸; Calero *et al.*, 2014 ▸), and can also be optimized and scaled via batch crystallization (Beale *et al.*, 2019 ▸; Wolff *et al.*, 2020 ▸). The identification of microcrystals from many different conditions in crystallization plates is not yet straightforward, as small microcrystals fall beyond what can be resolved by optical microscopes, and sometimes it is difficult to distinguish protein crystals from precipitate. Screening individual crystallization conditions using TEM is quite involved, as specimen preparation and cryo-sample handling can be quite complex (see also Section 3.3). It is therefore beneficial to develop a rapid sample-screening procedure in the MicroED workflow (Fig. 1 ▸). Potential crystal hits may be identified using UV fluorescence, SONICC or dynamic light scattering (DLS) (Judge *et al.*, 2005 ▸; Wampler *et al.*, 2008 ▸). These methods are promising for the identification of usually larger microcrystals. Another approach for screening is powder X-ray diffraction to confirm the crystallinity of the sample, which also enables the unit-cell dimensions to be measured from the diffraction patterns (Lanza *et al.*, 2019 ▸). However, the throughput has to be increased to screen many conditions. Furthermore, a highly concentrated sample is likely to be needed, which is typically not available in early-stage crystallization trials. Furthermore, cryogenic scanning electron microscopy (SEM) can provide information on the crystal morphology via imaging (Beale, Warren *et al.*, 2020 ▸), similarly to as in negative-stained TEM imaging (Sherman *et al.*, 1981 ▸).

**Figure 3:**
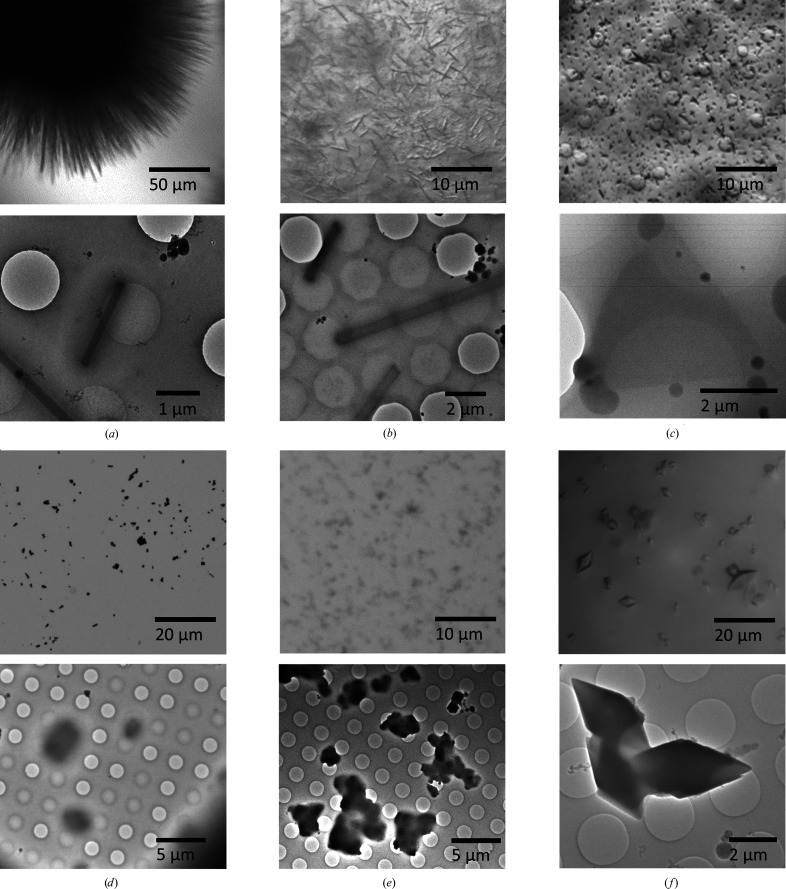
Typical crystal morphologies suitable for MicroED as seen under an optical microscope (top) and on the grid using TEM (bottom). (*a*) Needle-shaped nanocrystals of orthorhombic hen egg-white lysozyme; the crystals are 100–200 nm in thickness and several micrometres in length (Clabbers *et al.*, 2017 ▸; Xu *et al.*, 2018 ▸). (*b*) Needle-shaped dynamin GTPase microcrystals of 0.5–1.5 µm in diameter and several micrometres in length. (*c*) Triangular-shaped plate-like R2lox crystals; the crystals are less than 500 nm in thickness and a few micrometres in size (Xu *et al.*, 2019 ▸). (*d*) Fragments of large HCA II crystals; the fragments are 1–2 µm in size with a thickness of less than 500 nm (Fisher *et al.*, 2012 ▸; Clabbers *et al.*, 2020 ▸). (*e*) Fragmented crystals of tetragonal lysozyme; individual fragments are around 1 µm in size (Barends *et al.*, 2014 ▸). (*f*) Diamond-shaped R2c crystals of 2–15 µm in size. As the thinner edges of the crystals did not diffract well, MicroED data had to be collected from smaller crystals (Andersson & Högbom, 2009 ▸).

For MicroED, the crystal should preferably be as thin as still feasible in the direction parallel to the incident beam. Thus, crystal morphologies that are suitable for MicroED are typically needle- or plate-shaped (Fig. 3 ▸). Needle-shaped microcrystals are suitable since these are often very thin, making it possible to collect high-quality data (Clabbers *et al.*, 2017 ▸; Xu *et al.*, 2018 ▸). Using an electron beam with a small diameter, several data sets can be recorded along the length of the same crystal, similar to a helical scan in X-ray crystallography. By collecting data from crystals that lie parallel to the rotation axis, the deviation in thickness during continuous sample rotation is also minimized. However, it can be difficult to collect highly complete data from needle-shaped crystals when they have a large flat side causing a preferred orientation (Clabbers *et al.*, 2017 ▸; Xu *et al.*, 2018 ▸). Plate-shaped microcrystals are also often encountered and can be highly suitable for MicroED (Fig. 3 ▸). The overall diffracting signal becomes stronger when a larger area is illuminated, while the crystal is still thin enough in the direction parallel to the incident beam. However, as these crystals are flat, they often suffer from preferred orientation, and it is typically difficult to obtain data woth high completeness (Xu *et al.*, 2019 ▸). We recommend the collection of wedges of MicroED data over different mechanical tilt ranges in order to achieve reasonable data completeness. Alternatively, when only larger crystals are available these can be fragmented into smaller micrometre- and nanometre-sized crystal fragments by various means including vigorous pipetting, crushing with a glass spatula, vortexing with small beads or short pulses of ultrasonication (de la Cruz *et al.*, 2017 ▸; Fig. 3 ▸).

### 3.3. Specimen preparation   

**Figure 4:**
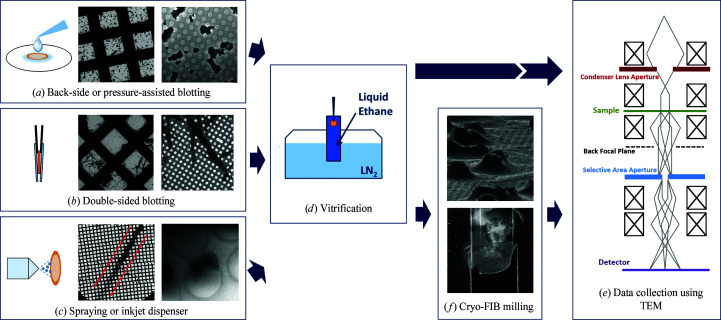
Schematic overview of the specimen preparation involved in MicroED. The aim of specimen preparation is to rapidly vitrify the hydrated protein crystals, while keeping a thin layer of vitrified ice around the crystals to protect them from the vacuum and electron beam radiation during MicroED data collection (Dubochet *et al.*, 1988 ▸; Shi *et al.*, 2016 ▸). (*a*) The protein crystal suspension is typically deposited on a 3 mm TEM grid (Quantifoil or lacey carbon). Prior to vitrification, any excess liquid can be removed by back-side blotting (Martynowycz & Gonen, 2020 ▸), pressure-assisted blotting (Zhao *et al.*, 2019 ▸) or (*b*) double-sided blotting (Shi *et al.*, 2016 ▸). (*c*) Alternatively, a small amount of crystal suspension can be sprayed onto a self-wicking grid, leaving a line of thin liquid trail covering approximately 50 grid squares, as highlighted by the red lines in the figure (Jain *et al.*, 2012 ▸; Klebl *et al.*, 2020 ▸). The concentration of the crystals in the suspension needs to be tuned to avoid clogging the nozzle of the inkjet. (*d*) After the excess liquid has been removed from the grid by any of the methods introduced above (*a*–*c*), the grid is then rapidly plunged into liquid ethane. (*e*) If the prepared specimen is of suitable thickness, MicroED data can be collected straight away using TEM. (*f*) Otherwise, cryo-FIB milling can be used to make thin crystalline lamella of the sample suitable for MicroED data collection (Duyvesteyn *et al.*, 2018 ▸; Zhou *et al.*, 2019 ▸; Martynowycz *et al.*, 2019*a* ▸,*b*).

There are several challenges when working with biological specimens in electron microscopy. Biological samples need to be conserved in their native hydrated state within the vacuum in the TEM column. Furthermore, radiation damage severely limits data collection. It is therefore critical that biological samples are vitrified and kept cooled at cryotemperatures during sample handling and data collection (Taylor & Glaeser, 1976 ▸; Dubochet *et al.*, 1988 ▸). Specimen preparation is thus a critical step in the workflow and commonly one of the major bottlenecks in studying macromolecules by electron microscopy. Vitrification using the deposit–blotting–plunging routine commonly used in cryo-EM specimen preparation for single-particle imaging can be adapted to prepare specimens suitable for MicroED (Dubochet *et al.*, 1988 ▸; Shi *et al.*, 2016 ▸). This process is typically automated using, for example, a Vitrobot (Thermo Fisher Scientific) setup, blotting away excess liquid from both sides of the EM grid (Fig. 4 ▸). After the excess liquid has been removed from the grid, the grid is then plunged into liquid ethane for rapid vitrification. The EM grid can thereafter be directly cryo-transferred to the TEM for MicroED data collection (Fig. 4 ▸).

Unfortunately, the majority of the crystals are usually removed from the grid when using double-sided blotting. Also, it is often not sufficient to remove highly viscous liquid. This becomes especially critical for protein crystals that are grown in highly viscous mother liquor, and even more so for microcrystals grown in LCP (see also Section 2.4). Alternatively, specimens can be prepared manually using back-side blotting and vitrification (Shi *et al.*, 2016 ▸; Martynowycz & Gonen, 2020 ▸; Fig. 4 ▸). Recently, a pressure-assisted back-side blotting method was introduced specifically for MicroED specimen preparation (Zhao *et al.*, 2019 ▸; Fig. 4 ▸). The method works for protein microcrystals grown in a wide range of buffer conditions, and the majority of the crystals are kept on the grid, minimizing sample loss. Alternatively, blotless specimen preparation uses a combination of self-wicking EM grids and spraying the crystal solution onto the grid, ensuring a thin layer of vitrified ice and minimizing sample loss (Jain *et al.*, 2012 ▸; Kontziampasis *et al.*, 2019 ▸; Rubinstein *et al.*, 2019 ▸; Tan & Rubinstein, 2020 ▸; Klebl *et al.*, 2020 ▸; Fig. 4 ▸). Although promising, blotless preparation methods have not yet been thoroughly tested for MicroED, and further experiments are required in order to evaluate how these methods can be applied to highly viscous sample media.

### 3.4. Cryo-FIB milling   

When crystals are slightly too large for MicroED but still too small for conventional X-ray crystallography, it is possible to prepare a thin crystalline lamella of the sample by cryo-focused ion beam (cryo-FIB) milling (Fig. 4 ▸; Duyvesteyn *et al.*, 2018 ▸; Zhou *et al.*, 2019 ▸; Martynowycz *et al.*, 2019*a* ▸). In cryo-FIB, the EM grids are firstly prepared by the methods introduced in Section 3.3. The grids are then cryo-transferred into an SEM equipped with a cryo-stage. The crystals of interest are identified by SEM imaging, while the milling is usually performed with a high-current Ga^+^ ion beam to create thin lamella. Irradiation damage at the surface of the crystals can be minimized by pre-coating the surface of the crystal with a platinum layer, increasing the electron conductivity and protecting the specimen from unintentional exposure to the ion beam (Martynowycz *et al.*, 2019*b* ▸). For the final stages of the milling process, a fine polishing step using a low-current ion beam can be performed to make a smoother crystal surface for MicroED data collection (Martynowycz *et al.*, 2019*b* ▸). Cryo-FIB makes it possible to collect MicroED data from crystals with a wider range of sizes and morphologies, even those crystals embedded in a thick layer of vitreous ice or highly viscous sample conditions such as LCP (Zhu *et al.*, 2020 ▸; Polovinkin *et al.*, 2020 ▸; Martynowycz, Shiriaeva *et al.*, 2020 ▸; Martynowycz, Khan *et al.*, 2020 ▸). Furthermore, creating large thin crystal lamella with controlled specimen thickness can improve the data quality. The ideal crystalline lamella is about 150–250 nm in thickness (Zhou, Luo & Li, 2019 ▸; Beale, Waterman *et al.*, 2020 ▸). Although highly promising, the protocol for cryo-FIB milling of protein crystals process can still be quite laborious and sample handling is not very robust.

## 4. Ongoing development and future opportunities   

### 4.1. Data quality   

In recent years, various protein structures have been determined by MicroED (Fig. 2 ▸). Although the structural models can be determined accurately, the standard crystallo­graphic intensity and model-quality statistics are typically worse than for X-ray diffraction. Uncertainties in the measured intensities can largely be attributed to random and systematic errors owing to data collection, data processing and instrumentation. These aspects, such as reliable and optimized rotation with minimal specimen drift, need to be addressed to increase the accuracy of the electron diffraction intensities (Yonekura *et al.*, 2015 ▸; Shi *et al.*, 2016 ▸; Smeets *et al.*, 2018 ▸). Furthermore, inelastic scattering increases the diffuse background and broadens the Bragg peaks in electron diffraction (Yonekura *et al.*, 2002 ▸, 2019 ▸; Clabbers & Abrahams, 2018 ▸; Latychevskaia & Abrahams, 2019 ▸). These effects can be mitigated by only collecting diffraction signals with zero energy loss using an energy filter, which reduces background noise and produces sharper Bragg spots (Yonekura *et al.*, 2002 ▸, 2019 ▸). This can be especially useful for the lower resolution reflections, as they may be shaded by the central beam and are difficult to extract owing to inelastic scattering events.

### 4.2. Dynamical scattering   

Whereas inelastic scattering can be discriminated by a measurable loss in energy, multiple elastic scattering (or dynamical scattering) cannot be separated from kinematic scattering. Because of dynamical scattering, weaker reflections on average will appear more intense. As a result, the first-order kinematic approximation that is used in structure refinement is no longer valid. An effective way to minimize dynamical scattering is to carefully select thin crystals (or thin crystalline lamella) for data collection. The crystal thickness ideally should not exceed much more than about 200 nm (Subramanian *et al.*, 2015 ▸; Clabbers & Abrahams, 2018 ▸; Latychevskaia & Abrahams, 2019 ▸; Zhou *et al.*, 2019 ▸; Beale, Waterman *et al.*, 2020 ▸). Dynamical scattering does not prevent protein structure solution and refinement, as even thicker crystals of up to about 500 nm in thickness have successfully been used for structure determination. Dynamical scattering is most severe when crystals are aligned on a zone axis. In MicroED, crystals are in a random orientation and generally not aligned along major zone axes, so that the dynamical effects are less. Currently, dynamical scattering may not be a key limitation in protein structure determination using the kinematic approximation. On the other hand, structure refinement using second-order dynamical diffraction theory is far more complex (Cowley, 1995 ▸; Dorset, 1995 ▸) and has so far only been applied to inorganic compounds and small molecules (Zandbergen, 1997 ▸; Jansen *et al.*, 1998 ▸; Palatinus *et al.*, 2017 ▸). Extending such refinement to macromolecules is far more complicated and computationally extensive, and an *a posteriori* scaling for dynamical electron scattering has been proposed (Clabbers *et al.*, 2019 ▸; Blum *et al.*, 2021 ▸).

### 4.3. Modelling the electrostatic potential   

Electrons are charged particles and interact with the electrostatic potential of the crystal (Cowley, 1995 ▸). However, the current programs used in crystallographic structure determination do not accurately model the electrostatic potential and charge distribution. Even though most software can work with atomic scattering factors for electrons, this does not take into account the asphericity and delocalization of electrons. Furthermore, the atomic scattering factors for electrons of (partially) charged ions differ significantly from those of neutral atoms, especially at lower resolution (Wu & Spence, 2003 ▸; Yonekura & Maki-Yonekura, 2016 ▸; Yonekura *et al.*, 2018 ▸). This can make it more difficult to resolve acidic side chains, as the atomic scattering factors fall off rapidly for negatively charged atoms at lower resolution (Mitsuoka *et al.*, 1999 ▸; Yonekura *et al.*, 2015 ▸). Therefore, the interpretation of MicroED data would benefit from accurately modelling the electrostatic potential (Grigorieff *et al.*, 1996 ▸; Mitsuoka *et al.*, 1999 ▸; Wu & Spence, 2003 ▸; Yonekura *et al.*, 2015 ▸). This would have extensive implications for protein crystallography, enabling the visualization of the charge states of atoms and the electrostatic potential in covalent bonds and lone electron pairs, providing novel insights into protein function and binding interactions.

### 4.4. Isomorphous replacement   

To date, all structural models of proteins determined by MicroED have been phased using molecular replacement. Direct methods can be used for electron diffraction data (Dorset & Hauptman, 1976 ▸; Dorset, 1995 ▸). Direct methods have been applied for the *ab initio* phasing of short peptide fragments (Sawaya *et al.*, 2016 ▸; Gallagher-Jones *et al.*, 2018 ▸), but macromolecules are typically too complex and lack a favourable data-to-parameter ratio for such methods. It has been demonstrated that fragment-based molecular replacement is feasible using MicroED data at better than 2.0 Å resolution (Richards *et al.*, 2020 ▸). However, *de novo* phasing of protein structures using electron diffraction has not yet been achieved. The anomalous signal may be too insignificant for experimental phasing using anomalous dispersion (Cowley, 1995 ▸). Experimental phasing using isomorphous replacement is promising but complicated. The expected difference in signal between the native crystal and a heavy-atom derivative is lower than in X-ray diffraction, as the atomic scattering factors of the heavier atoms differ less from the lighter atoms in electron diffraction. Furthermore, non-isomorphism and inaccuracies of the measured intensities further complicate isomorphous replacement (Crick & Magdoff, 1956 ▸).

### 4.5. Phasing by TEM imaging   

In 2D electron crystallography, reconstructions from TEM images can be combined with high-resolution electron diffraction patterns for structure determination (Unwin & Henderson, 1975 ▸; Mitsuoka *et al.*, 1999 ▸; Gonen *et al.*, 2005 ▸). For inorganic samples, atomic positions can be resolved from electron micrographs of 3D crystals (Hovmöller *et al.*, 1984 ▸; Downing *et al.*, 1990 ▸; Dong *et al.*, 1992 ▸). After obtaining initial atomic positions and crystallographic structure-factor phases, the reflection intensities extracted from electron diffraction patterns can be used for refinement of the atomic positions and extension to higher resolution (Weirich *et al.*, 1996 ▸; Zandbergen, 1997 ▸; Jansen *et al.*, 1998 ▸; Zou *et al.*, 2003 ▸). These routines have not successfully been applied to phase macromolecular structures from 3D crystals thus far (Nederlof, Li *et al.*, 2013 ▸; Peck *et al.*, 2020 ▸). Phasing of 3D macromolecular crystals is complicated by crystal thickness, breaking the weak phase object approximation, and corrections for the contrast transfer function (CTF), as well as difficulty in finding a common origin.

### 4.6. Serial electron diffraction   

In macromolecular crystallography, the total electron dose limits the maximum attainable resolution and data quality. Using the continuous-rotation method in MicroED, the critical accumulated dose is spread out over an entire tilt series to sample a substantial wedge in reciprocal space. In contrast, in SFX only single snapshots are recorded from tens of thousands of individual crystals (Schlichting, 2015 ▸; Spence, 2017 ▸). With similar size requirements for protein microcrystals, MicroED and SFX can complement one another (Zatsepin *et al.*, 2019 ▸; Wolff *et al.*, 2020 ▸). The same principle as in SFX can also be applied in TEM as serial electron diffraction (SerialED; Smeets *et al.*, 2018 ▸; Bücker *et al.*, 2020 ▸). Using SerialED, a single electron diffraction pattern can be taken from one crystal in a single exposure (Smeets & Wan, 2017 ▸; Wang *et al.*, 2019 ▸). Alternatively, dose-fractioning can be used (Bücker *et al.*, 2020 ▸). Here, still electron diffraction patterns are collected at a rapid rate at a constant electron dose rate. Based on the critical dose of the crystal, an appropriate number of frames are summed together to form a single serial electron diffraction pattern. For HEWL microcrystals, a critical dose of 2.6 e^−^ Å^−2^ was found to have an optimal signal-to-noise ratio (Bücker *et al.*, 2020 ▸).

In SerialED, only a single still diffraction pattern of a randomly orientated crystal is acquired, lacking information about unit-cell parameters owing to the large Ewald sphere. This limitation can be overcome by automation, collecting and integrating data from many thousands of randomly oriented crystals (White *et al.*, 2013 ▸; Bücker *et al.*, 2020 ▸). SerialED and MicroED can also be combined to achieve high-throughput automated structural analysis of macromolecules (Wang *et al.*, 2019 ▸). In typical SerialED data collection, an atlas of the grid is generated. Data-collection software automatically identifies the crystals and generates a table of crystal positions. By utilizing stage movement and beam shift, diffraction patterns are collected from each individual crystal. With a sophisticated alignment protocol or improved hardware, it should be possible to collect complete or almost complete 3D ED data. Automated pipelines can also be implemented to process MicroED data on the fly through data-streaming setups similar to those used in single-particle analysis. Together with a robust specimen-preparation protocol, high-throughput MicroED would bring new opportunities not only to the rapid structure determination of macromolecules but also to fragment-based screening for drug discovery.

## 5. Conclusions   

Here, we illustrate the various achievements made by MicroED in the past years, and discuss the novel opportunities that it may bring for structural biology. Recent successes include determining increasingly challenging structures, resolving ligand-binding interactions and enabling structure determination of membrane proteins from microcrystals embedded in lipidic mesophases. The field is still evolving, and improvements in specimen preparation, optimization of TEM hardware and accurate modelling of the electrostatic potential are needed. Even more so, *de novo* phasing through experimental phasing and/or high-resolution imaging may be realized in the years to come. We anticipate that with these advances, MicroED will play an ever more important role in macromolecular crystallography.
